# Colchicine to Prevent Sympathetic Denervation after an Acute Myocardial Infarction: The COLD-MI Trial Protocol

**DOI:** 10.3390/medicina57101047

**Published:** 2021-09-30

**Authors:** Fabien Huet, Quentin Delbaere, Sylvain Aguilhon, Valentin Dupasquier, Delphine Delseny, Richard Gervasoni, Jean-Christophe Macia, Florence Leclercq, Nidal Jammoul, Sandra Kahlouche, Sonia Soltani, Fanny Cardon, Anne-Marie Dupuy, Jean-Paul Cristol, Denis Mariano-Goulart, Myriam Akodad, Nicolas Nagot, François Roubille

**Affiliations:** 1 Department of Cardiology, Montpellier University Hospital, 34295 Montpellier, France; f-huet@chu-montpellier.fr (F.H.); quentin.delbaere@hotmail.fr (Q.D.); sylvain.aguilhon@gmail.com (S.A.); valentin.dupasquier@wanadoo.fr (V.D.); d-delseny@chu-montpellier.fr (D.D.); r-gervasoni@chu-montpellier.fr (R.G.); maciajc@gmail.com (J.-C.M.); f-leclercq@chu-montpellier.fr (F.L.); nidal.jammoul@gmail.com (N.J.); s-kahlouche@chu-montpellier.fr (S.K.); s-soltani@chu-montpellier.fr (S.S.); akodadmyriam@gmail.com (M.A.); 2 PhyMedExp, INSERM, CNRS, Université de Montpellier, 34295 Montpellier, France; 3 Biostatistics and Research Unit, CHU Montpellier, Montpellier University Hospital, 34295 Montpelier, France; fanny-cardon@chu-montpellier.fr (F.C.); n-nagot@chu-montpellier.fr (N.N.); 4 Department of Biochemistry, Montpellier University Hospital, 34295 Montpellier, France; am-dupuy@chu-montpellier.fr (A.-M.D.); jp-cristol@chu-montpellier.fr (J.-P.C.); 5 Department of Nuclear Medicine, Montpellier University Hospital, 34295 Montpellier, France; d-mariano_goulart@chu-montpellier.fr

**Keywords:** colchicine, sympathetic innervation, myocardial infarction, heart rate variability, nuclear imaging

## Abstract

Inflammatory processes are deeply involved in ischemia-reperfusion injuries (IRI) and ventricular remodelling (VR) after a ST-segment elevation myocardial infarction (STEMI). They are associated with clinical adverse events (heart failure and cardiovascular death) adding damage to the myocardium after reperfusion. Moreover, acute myocardial infarction (AMI) induces a local sympathetic denervation leading to electrical instability and arrythmia. Colchicine, a well-known alkaloid with direct anti-inflammatory effects, was shown to reduce the myocardial necrosis size and limit the VR. In a recent proof of concept study, colchicine appears to prevent sympathetic denervation in a mice model of ischemia/reperfusion, but not in the necrosis or in the border zone areas. The Colchicine to Prevent Sympathetic Denervation after an AMI study (COLD-MI) is an ongoing, confirmative, prospective, monocentre, randomized, open-label trial. The COLD-MI trial aims to evaluate the intensity of sympathetic denervation after AMI and its potential modulation due to low dose colchicine. Sympathetic denervation will be noninvasively evaluated using single-photon emission computed tomography (SPECT). After a first episode of STEMI (Initial TIMI flow ≤ 1) and primary percutaneous coronary intervention (PPCI), patients will be randomized (n = 56) in a 1:1 ratio to either receive colchicine or not for 30 days. The primary end point will be the percentage of myocardial denervation measured by 123I-metaiodobenzylguanidine (123I-MIBG) SPECT at a 6-month follow-up. The main secondary end points will be basic ECG parameters (QRS duration, corrected QT) and HRV parameters from a 24 hour-recording Holter at 1- and 6-months follow-up. Results from this study will contribute to a better understanding of the cardioprotective effect of colchicine after AMI. The present study describes the rationale, design, and methods of the trial.

## 1. Introduction

Early and successful myocardial reperfusion is the most effective strategy for reducing the size of myocardial infarction due to acute myocardial infarction (AMI). However, restoring the blood flow acts as a double-edged sword, inducing also lesions known as ischemia-reperfusion injuries (IRI) [1]), including no-reflow lesions, myocardial sideration and necrosis. Immediately after reperfusion, inflammatory signals including cytokines such as interleukin (IL)-1β and IL-6 are liberated. Simultaneously, innate immunity is activated and leads to the recruitment of inflammatory cells with sequential implication of neutrophils, mononuclear cells, dendritic cells and lymphocytes. Neutrophils are strongly involved in initiation and resolution of inflammation, angiogenesis and infarct recovery, whereas T lymphocyte and dendritic cells mostly contribute to ventricular remodelling (VR) [2]. Inflammation is deeply involved during AMI leading to deleterious effects at the very onset of the reperfusion.

Sympathetic control of the heart under normal conditions occurs primarily via norepinephrine acting on β adrenergic receptors which allow myocytes to adapt to increased cardiac demands during stress or exercise, contributing to homeostasis [3]. In a myocardial infarcted area, a negative remodelling of the sympathetic nervous system with local sympathetic denervation has been previously demonstrated [4]. Several clinical studies suggest that sympathetic denervation in the area subjected to potential ischemia, the so-called “area at risk” (AAR) and in the infarcted area leads to an electrical instability and predicts the probability of serious ventricular arrhythmia (VA) [5,6]. For clinical purposes, we have shown that sympathetic denervation in the AAR can be rigorously evaluated in patients with 123I-metaiodobenzylguanidine (123I-MIBG) single-photon emission computed tomography (SPECT) [7,8]. Consistently, heart rate variability (HRV) appears to be a key tool in clinical practice for predicting arrhythmias and mortality in patients after AMI [9,10].

As inflammatory cell infiltration is the first step of local inflammation, it has become a therapeutic target and the decrease of inflammatory processes has been shown to limit IRI in preclinical studies. Colchicine is a mitotic spindle poison with direct anti-inflammatory effects, reducing the migration of neutrophils in ischemic myocardial injured tissues [11,12,13], reducing myocardial necrosis and limiting VR in a murine model [14]. More recently, colchicine has also been shown to protect from myocardial denervation in remote areas and decrease HRV in mice [15], but not in the border zone or in the necrosis area. Its benefit for patients remains unknown until now. The promising impact of colchicine in clinical practice has been demonstrated in two large international trials: first in acute coronary syndromes [16] and then in stable coronary artery disease [17] even though, paradoxically, the underlying protective pathways remain to be fully understood [18].

Thus, the objective of this study was to explore colchicine’s impact on myocardial denervation following reperfused AMI by evaluating the remodelling of cardiac nerves using the 123I-MIBG SPECT.

## 2. Materials and Methods

### 2.1. Trial Design

The COLD-MI trial is a randomized, monocentric, open-label trial. Open labelled design has been chosen because colchicine has already been shown to increase prognosis in a large population of patients, and because the MIBG analysis has a low interobserver variability and will be analysed blind.

Because colchicine has already been demonstrated to be effective in reducing events after MI, it was not deemed mandatory to use a control-based design.

Following signatures of informed consent, 56 subjects meeting all inclusion criteria and no exclusion criteria will be randomized to receive either colchicine (dose according to protocol) or not (1:1 allocation ratio) for 30 days. A follow-up visit will occur at 1 and 6 months after randomization. The study schedule is presented in Table 1.

Patients will receive standard medical care according to the European Society of Cardiology Guidelines for the management of acute myocardial infarction in patients presenting with ST-segment elevation [19]. The protocol was approved by the Institutional Review Board and the Agence Nationale de Sécurité du Médicament et des Produits de Santé (ANSM). The trial medication was provided by the in-hospital pharmacy, which had no role in the design or conduct of the trial or in the preparation or review of the manuscript. The trial aims to evaluate the impact of colchicine on:

- Sympathetic denervation at 6 months after an AMI;
- Heart rate variability at 1 and 6 months after an AMI.

### 2.2. Trial Population

Investigators will obtain informed consent from patients who volunteer to participate in the trial prior to the administration of the studied treatment. The patient will be “enrolled” into the study at the time an informed consent is provided.

Inclusion criteria are:

- Adult patients;
- Presenting with a clinical episode of STEMI (chest pain and ECG abnormalities (12 leads) consistent with the diagnosis of AMI: elevation of the ST segment on at least 2 contiguous branches, or appearance of a recent left branch block);
- Chest pain lasting less than 12 h;
- Coronary occlusion on initial angiography (TIMI flow of the causal artery of the infarction at 1 or 0);
- Eligible for a revascularization procedure by percutaneous coronary angioplasty.

Exclusion criteria are:

- Any history of myocardial infarction prior to the current episode;
- Severe hepatic or renal dysfunction (known GFR ≤30mL/min);
- Chronic treatment with Colchicine (mainly Familial Mediterranean Fever);
- Chronic treatment with a potent CYP3A4 inhibitor or P-glycoprotein inhibitor in patients with kidney or liver failure;
- Any history of severe reaction or known severe intolerance to colchicine;
- Association with macrolides (except spiramycin) or with pristinamycin;
- Hemodynamic instability (need for amines for more than 24 h, assistance circulatory system) at the time of inclusion;
- Disorders of conscience compromising informed consent;
- Swallowing disorders and major digestive disorders (chronic diarrhoea, inflammatory bowel disease, etc..), gastrointestinal tract disease such as uncontrolled ulcerative colitis or Crohn’s disease, with immunosuppression;
- Bone marrow aplasia, active chronic inflammatory disease, chronic active infection;
- Active cancer, bone marrow aplasia;
- Chronic active infection or recent sepsis (7 days);
- Chronic treatment (for more than 6 months) with corticosteroids or NSAIDs or repeated high doses of less than 7 days, corresponding to a corticoid dose of >1mg/kg/d for more than 3 consecutive days or ibuprofen >600 mg/d for more than 3 days) or treatment with potent CYP3A4 inhibitors such as cyclosporin, verapamil, inhibitors of proteases boosted by ritonavir and telaprevir;
- Contraindication to an examination of the study (scintigraphy, …);
- Legal protection measure (guardianship, curatorship);
- Participation in another clinical trial.

### 2.3. End Points

The primary end point is the percentage of myocardial denervation measured by 123I-MIBG SPECT at 6-months follow-up.

Secondary ends points are described in the following sections:

#### 2.3.1. Biological Assessments

- Variations in the levels of Nerve Growth Factor, C-reactive Protein (CRP) and Sst2 in plasma between hospitalisation, 1- and 6-months follow-up;
- Assessment of infarct size with area under the curve of the creatine kinase and troponin during the hospitalisation (day 1 to day 5).

#### 2.3.2. Imaging Assessment

- SPECT imaging at 6 months follow-up:
  ○ heart/mediastinum ratio (quantitative index in cardiac imaging 123 I-MIBG SPECT),
  ○ isotopic LVEF;
- TTE at 1 and 6 months:
  ○ Left ventricular ejection fraction (LVEF) in percent;
  ○ Global longitudinal strain imaging in percent.

#### 2.3.3. Rhythmic Evaluation

- ECG parameters (QRS duration, corrected QT) at 1- and 6-months follow-up;
- HRV parameters on a 24 hour-recording Holter at 1- and 6-months follow-up. In a 24h Holter monitoring, HRV parameters are measured. According to the Task Force of the European Society of Cardiology and the North American Society of Pacing and Electrophysiology [10], we decided to assess both time and frequency domain measures:
  ○ Time-domain measures are represented by the Standard Deviation of NN intervals (SDNN), the Root mean square of successive RR interval differences (RMSSD) and the Baseline width of the RR interval histogram (TINN). All these measures are expressed in milliseconds.
  ○ Frequency-domain measures divide the heart rate into 4 bands. We use the Very Low (VLF), Low (LF) and High (HF) Frequency bands, expressed in absolute power (ms squared divided by cycles per second);
- Number of premature ventricular contraction (PVC) at 1- and 6-months follow-up;
- Number of bursts (2 or 3 PVC) at 1- and 6-months follow-up;
- Number of episodes of supraventricular or ventricular tachycardia (defined by >3 PVC) at 1- and 6-months follow-up.

#### 2.3.4. Exploratory Clinical Evaluation

- Number of adverse events;
- Number of all-cause hospitalizations, hospitalization for heart failure or death.

## 3. Intervention

**Figure 1:**
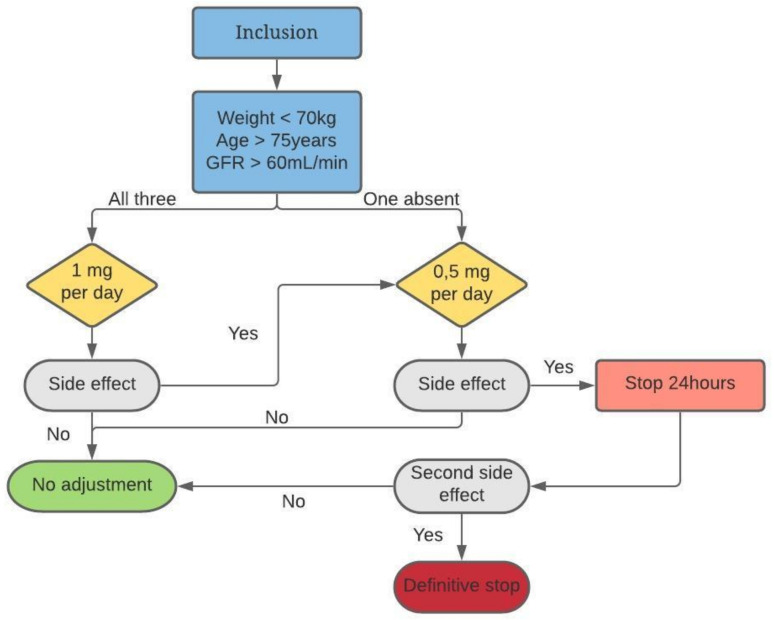
Algorithm of dose adjustment in case of side effects. GFR: Glomerular Filtration Rate.

Medication will be provided as 1 mg colchicine tablets. We use Colchicine Opocalcium 1mg, with 15 tablets per box, one per day for 30 days, orally. A detailed set of dispensing instructions will be included with the drug shipment. Study medication will be dispensed by the designated pharmacist or a qualified investigator. At randomization, treatment will be initiated within 48 h and patients will receive colchicine tablets or none for 30 days. Patients will be instructed to take tablets once daily according to the detailed set of dispensing instructions. At the follow-up visit at 1 month, empty shelves will be recovered to assess patient compliance. Because of possible side effects, mostly digestive disorders, doses will be adapted according to the tolerance as indicated in Figure 1.

Drug safety will be assessed by an evaluation of types, frequencies, severities and duration of any reported adverse events. Patients will be monitored for signs and symptoms of drug toxicity.

## 4. Data Collection and Analysis

All data recorded for the purpose of this research are subject to computerised processing under the responsibility of the Montpellier University Hospital, the promoter, in compliance with law n°78-17 of 6 January 1978 as amended and the Regulation on the protection of individuals with regard to the processing of personal data and on the free movement of such data, and repealing directive 95/46/EC (general regulation on data protection or RGPD). Based on preliminary data showing a mean of 24% of the myocardial denervation zone (trigger zone) with a standard deviation of 17.5, the recruitment of 50 patients will demonstrate an absolute reduction of 10% of the mean, with a power of 80%. This 10% reduction of the denervated area corresponds to the minimal clinical benefit expected to consider the use of colchicine in this indication. As all patients were routinely followed up at 6 months, the expected rate of lost to follow-up patients at 6 months was estimated at 10%, i.e., a total of 56 patients would be included in the trial. Study data were collected using the CSOnline 7.5.720.1 (Ennov Clinical) software.

Analyses will be performed using the SAS software (SAS institute, Cary, NC, USA). Normally distributed data will be presented using mean and standard deviation, and non-normally distributed data will be presented using median and interquartile range. Categorical data will be presented as count (%). Continuous variables concerning patient characteristics and treatment will be performed using Student’s t-test, or Wilcoxon-Mann–Whitney test (in case of non-normal distribution). Categorical variables will be compared using χ2 test, or Fisher’s exact test (in case of unmet χ2 conditions). Survival data will be presented using Kaplan–Meier curves. Univariate and multivariate analyses will be performed using linear regression. Follow-up data will be analysed with log-rank test and survival analysis.

## 5. Discussion and Conclusions

Authors should discuss the results and how they can be interpreted from the perspective of previous studies and of the working hypotheses. The findings and their implications should be discussed in the broadest context possible. Future research directions may also be highlighted.

This study aims to evaluate the effectiveness of colchicine on myocardial denervation and the risk of arrythmias. This project translates from preclinical study to bedside. Indeed, our team demonstrated in mice that colchicine improved HRV and reduced cardiac sympathetic denervation in the remote area in a mice model of ischemia reperfusion. In vivo telemetry was performed 24 h and 6 days after the AMI. ECG analyses have shown that colchicine significantly reduces QRS and QT duration in comparison to placebo. HRV parameters were also improved in colchicine group, as they proved to be strong predictors of adverse events after an AMI [20].

Colchicine may therefore regulate electrophysiological properties at the cellular level. Due to its action on microtubule depolymerization, it was proposed that colchicine could activate several G-proteins. These G-proteins might increase Acetylcholine (Ach)-dependent K+ channels and L-type Ca^2+^ current conductance. Altogether, this could explain the moderation of QT interval prolongation after an AMI [21]. Finally, colchicine prevented denervation in the remote area on histological sections, which had already been shown to increase the risk of sudden death [22] and be correlated to myocardial remodelling after an AMI.

The main obstacle on the translation from preclinical results to bedside is the multiple therapeutic management of AMI. If mice only receive colchicine in infarction models, our patients already benefit from a drug cocktail shown to be safe and effective [19,23]. Indeed, the incidence of VA has strongly declined over recent decades, and beta-blockers in particular have already been shown to reduce VA after an AMI [24]. This might partly explain why significant clinically relevant results are difficult to obtain in clinical trials. However, 6–8% of patients still develop haemodynamically significant ventricular tachycardia or fibrillation during the acute phase, and 20% of these died because of this arrythmia [25].

Colchicine has already demonstrated its effectiveness in post-AMI prevention. In the large Colchicine Cardiovascular Outcomes Trial (COLCOT) study, colchicine reduced the primary efficacy end point (composite of death from cardiovascular causes, resuscitated cardiac arrest, myocardial infarction, stroke, or urgent hospitalization for angina leading to coronary revascularization) by 23% [16]. Moreover, early initiation of colchicine within the 3 days after AMI was associated with a reduction of 48% in the risk of the primary endpoint, suggesting the positive impact of treatment on the immediate inflammatory response [26].

Translating myocardial innervation to AAR assessment is possible in clinical practice. 123I-MIBG SPECT has been shown to be a reliable imaging technique to assess myocardial denervation and AAR. However, it remains less sensitive than the histological exploration used in preclinical studies.

Our study will probably help to provide additional evidence that colchicine would bring a real benefit in VR occurring after an AMI. For patients, benefits would be a reduction in hospitalization, severe VA and mortality due to rhythmic disorders. Moreover, it is a long-established medicine (first used in 1820) and is generic and low cost. Widely used in rheumatology’s field, colchicine is also given in pericarditis treatment to avoid recurrence due to chronic inflammation. All this allows us to have a solid knowledge of this long-term treatment and especially its side effects. Indeed, in the large COLCOT trial, there were no significant differences between colchicine and placebo groups for gastro-intestinal events, infection or septic shock as reported in the Anti-inflammatory Therapy with Canakinumab for Atherosclerotic Disease (CANTOS) trial [27]. No serious adverse event of myopathy linked to colchicine, despite the use of statins in 99% of the patients in the trial, was reported either.

Last but not least, the results from the Colchicine for Left Ventricular Remodelling Treatment in Acute Myocardial Infarction (COVERT-MI) trial [28] should be known by the end of 2021. In COVERT-MI, colchicine was supposed to reduce the infarct size in AMI. The COLD-MI trial will provide mechanistic explanations for this trial and others.

Colchicine is therefore gradually finding its place in the therapeutic arsenal of coronary disease, both in the acute phase by reducing the size of the necrosis and its rhythmic complications and in the chronic phase by slowing down the formation of atherosclerosis. Studies will continue to explore the risk/benefit ratio in order to validate the place of colchicine in our practices.

## Figures and Tables

**Table 1:** Timetable of Visits and Procedures. MRI: Magnetic Resonance Imaging, ECG: Electrocardiogram. AE: Adverse Event.

Visit	Inclusion	Visit 1	Visit 2	Visit 3	Data Collection	Visit 4	Visit 5	Visit 6
Date	Day 0	Day 1	Day 2	Day 3	Day 4, 5	Out of hospital	1 month ± 1 week	6 month ± 1 week
Informationa and written consent signature	X							
Randomization		X						
Clinical examination	X					X	X	X
Review concomitant treatment	X	X	X	X	X	X	X	X
Blood sample ^(1)^	X	X	X	X	X ^(3)^	X	X	X
Specific analysis and biological collection ^(2)^	X *	X *	X *	X *			X *	
Urine pregnancy test (only women of childbearing potential)	X *							
Transthoracic echocardiography	X					X	X	X
Cardiac MRI	X							
^123^I-MIBG SPECT								X *
Holter ECG 24h						X	X	X
Rest ECG	X					X	X	X
AE collection	X *	X *	X *	X *		X *	X *	X *
Study medication dispensing		X *	X *	X *	X *	X*	X *	
Study medication return							X *	

* project specific; ^(1)^ Usual blood sample (20mL): urea, creatinine, liver function, complete blood count, lipid profile, glycaemia, Hba1c, C-reactive Protein (CRP), NTproBNP, hs-cTnT. ^(2)^ 16mL blood sample: Troponin, IL1β and IL6, SST2, collagen markers; ^(3)^ only CPK, CRP, hs-cTnT and myoglobin.
